# Citizen Science and Community Engagement in Tick Surveillance—A Canadian Case Study

**DOI:** 10.3390/healthcare6010022

**Published:** 2018-03-02

**Authors:** Julie Lewis, Corinne R. Boudreau, James W. Patterson, Jonathan Bradet-Legris, Vett K. Lloyd

**Affiliations:** Department. Biology, Mount Allison University, Sackville, NB E4L 1G7, Canada; jlewis@mta.ca (J.L.); crboudreau@mta.ca (C.R.B.); jpatterson@mta.ca (J.W.P.); jhbradetlegris@mta.ca (J.B.-L.)

**Keywords:** tick surveillance, Lyme disease, citizen science, community partnership, crowdsourcing, public health

## Abstract

Lyme disease is the most common tick-borne disease in North America and Europe, and on-going surveillance is required to monitor the spread of the tick vectors as their populations expand under the influence of climate change. Active surveillance involves teams of researchers collecting ticks from field locations with the potential to be sites of establishing tick populations. This process is labor- and time-intensive, limiting the number of sites monitored and the frequency of monitoring. Citizen science initiatives are ideally suited to address this logistical problem and generate high-density and complex data from sites of community importance. In 2014, the same region was monitored by academic researchers, public health workers, and citizen scientists, allowing a comparison of the strengths and weaknesses of each type of surveillance effort. Four community members persisted with tick collections over several years, collectively recovering several hundred ticks. Although deviations from standard surveillance protocols and the choice of tick surveillance sites makes the incorporation of community-generated data into conventional surveillance analyses more complex, this citizen science data remains useful in providing high-density longitudinal tick surveillance of a small area in which detailed ecological observations can be made. Most importantly, partnership between community members and researchers has proven a powerful tool in educating communities about of the risk of tick-vectored diseases and in encouraging tick bite prevention.

## 1. Introduction

Lyme borreliosis, also known as Lyme disease, is the most common tick-borne disease in North America and Europe [1,2]. The disease is initiated by an infection with a member of at least 19 species of bacteria in the *Borrelia* genus known as the Lyme borreliosis group or *Borrelia burgdorferi sensu lato* [3,4]. If undetected and untreated, Lyme borreliosis can cause debilitating and, in some cases, fatal, multisystem symptoms [5,6,7]. 

In North America, *Ixodes scapularis* is the primary vector in the eastern and central parts of the continent, and *Ixodes pacificus* is prevalent in in the western regions, although *Ixodes cookei*, *Ixodes angustus*, and *Ixodes muris* have also been found to be vectors, and other species are potential vectors [8,9,10,11]. In Europe, the *Ixodes ricinus* species group is the primary vector [1,3]. Tick populations are expanding their range in response to climate change in North America, and this has brought them to Canada [12]. However, the prediction of new areas of population expansion is challenging because of the constant seeding of adventitious ticks introduced by migratory birds and mammals [13,14,15,16]. The survival of these ticks in either transient or small local populations, only some of which may proliferate into large, established “endemic” tick populations, is difficult to detect, yet important, as even small and transient populations pose a health risk to those living in those areas. 

As ticks are expanding their range, the risk to public health has mobilized considerable resources to generate Lyme borreliosis risk maps and models [17,18,19]. These maps and models draw, in various measures, upon passive tick surveillance—ticks collected on companion animals and humans—or field collection of ticks, also known as active surveillance or tick dragging. In addition, case reports from humans, environmental factors such as climate, biogeography, distribution of the wildlife species needed to sustain tick and *Borrelia* populations, and canine Lyme seropositivity studies have been used to predict the risk of Lyme borreliosis [18]. While these risk models aim to predict areas where tick populations may establish, with the attendant evaluated risk of tick-vectored diseases, these models all require field validation, most frequently by active surveillance. Active surveillance, collecting ticks on a cloth dragged through potential tick habitat, is widely recognized to suffer from being a low-sensitivity method of tick detection. For example, Koffi et al. (2012) [19] reported that only 60% of the predicted tick high-risk areas yielded ticks upon field sampling. Similarly, a retrospective study of active surveillance of areas that subsequently became endemic showed only 50% sensitivity [20]. Thus, the low sensitivity of this form of surveillance is useful when defining tick endemic areas, large areas with high tick density, but is not well suited for identifying areas where tick populations are emerging. Additionally, field sampling is a logistically complex and expensive process, and, as a result, field teams generally only visit a site once. If the weather, day, time, or any of a host of other factors is not suitable, ticks may not be recovered. It is here that citizen science can play an important role by mobilizing citizens to monitor their own neighbourhoods and regions. 

Citizen science involves engaging members of the general community in order to “crowdsource” data acquisition. The value of citizen science for researchers lies in the capacity for a tremendous expansion in data acquisition capacity. Universities are well positioned to engage in such community-centered research initiatives as many already have active community-engagement policies and practices; the same rational applies to public health researchers. From the community perspective, citizen science allows members of the public to not only explore an intrinsic interest in the natural world, but also engage in research relevant to their own health and that of their families and community members. When individuals are engaged in scientific research, as they are in citizen science projects, there is a heightened trust in science leading to personal empowerment, which underlies changes in behaviour that are needed to adapt to the changing environmental risk. The value of the citizen science approach has been appreciated, and citizen science has been extensively incorporated into ecological studies, but much less so in public health initiatives [21,22].

Passive tick surveillance involves members of the public, veterinary or humanmedical professionals submitting ticks for study. This type of “crowdsourcing” of ticks is highly effective for surveillance [17,23] as well as in providing ticks for a tick bank, bioclimatic modeling, or other purposes, as exemplified by the study of Laaksonen et al. (2017) [23], in which nearly 20,000 crowdsourced ticks were used to map changed tick distributions and new tick-vectored pathogens in Finland. If such initiatives return the results of tick pathogen testing to the donor, both partners benefit. However, with increased community involvement, even greater engagement and mutual benefit is achieved [21,22,24]. 

One way to increase community participation is by partnering with community volunteers in active tick surveillance. Members of the public are in a position to intensively monitor the same site, for example a backyard, favorite park, or school playground, over one or many seasons. For example, Seifert et al. (2016) [24] described the success of a program of tick education implemented in rural high schools, a tribal school, and a correctional facility that involved training volunteers in active tick surveillance. This project demonstrated that this active participation increased student knowledge of tick biology, awareness of tick bite prevention strategies, recognition of common signs and symptoms of Lyme disease, and student interest in science. All of these outcomes are highly desirable from the public health, medical, and societal perspectives. On a national scale, Garcia-Marti et al. (2017) [25] reported on the impressive results of a large study in Holland. In this project, trained volunteers conducted active surveillance, producing extensive and detailed collection records composed of over 3000 observations at 15 sites over nine years. This large and comprehensive dataset allowed geographic and spaciotemporal mapping of tick populations and pathogens at the national level. While traditional public health active surveillance initiatives are constructed around a standardized research methodology, as demonstrated by Garcia-Marti et al. (2017) [25], the variability in collection methodology implicit in citizen science initiatives is still compatible with highly effective public health surveillance. 

The research question addressed here focuses on the relative strengths and advantages of academic, public health, and community-driven tick surveillance efforts. We approached this question by comparing the outcomes of each of these surveillance approaches, conducted during the same time period and in the same region. The volunteer community surveillance initiatives generated the greatest number of ticks, over a period of several years, at virtually no cost. While non-conventional and diverse methodology was used, these community tick collections provide detailed information on tick seasonal activity, abundance, density, infection rate, ability to overwinter, and similar biological factors, data not otherwise readily attainable. Most importantly, this initiative resulted in extensive community-based peer education efforts. Thus, partnerships between community volunteers and researchers promotes both research and education on the health risk posed by ticks.

## 2. Materials and Methods

### 2.1. Tick Collection—Academic Researchers

Field collection of ticks was performed by “tick dragging”. A piece of animal scent-treated (wet dog or sheep) flannel, sourced from a thrift store, one square meter in dimension with solid wooden rods at each end and a rope at the front for pulling, was slowly dragged on the ground at a pace of approximately 8.6 m/min. Every ten paces, the sheets were checked for ticks. Each site was sampled for approximately 3 person-hours. Field workers wore protective clothing and performed tick checks. The choice of the surveillance locations was determined on the basis of records from passive tick surveillance initiatives (Lewis and Lloyd, unpublished) and canine seroprevalence studies [26], and of information on tick encounters from community leaders, veterinarians, and Lyme advocacy groups. Tick dragging sites generally included tall grass, areas with leaf litter, and broken woods. Ticks removed from the tick drags or the field workers were placed in a labeled container for same-day transport to the laboratory where species identification and DNA extraction took place. No animal care or environmental certification was required for these collections.

### 2.2. Tick Collection—Public Health 

Although the criteria for site selection differed from the academic study, field tick collection was performed in essentially the same manner. Each site was sampled once by the same researcher, sites were 10,000 m^2^ in size, and ticks were collected by dragging a one-meter flannel through vegetation, as described by Gabriele-Rivet et al. (2015) [27].

### 2.3. Tick Collection—Community Members

For the recruitment of community volunteer researchers, members of the community or municipal leaders contacted the senior author of this study for information on tick surveillance. Tick collections by community members were in some cases conducted with the academic researchers, using a standard approach. Those citizen scientists who joined the tick drags were instructed on personal protective clothing and in how to do tick checks. In other cases, tick collection was conducted independently and varied in time spent, area surveyed, and method employed. The same information on tick repellents, suitable clothing, and tick checks was conveyed to those collectors who approached the academic researchers with pre-existing tick collections. The Nova Scotia collection was obtained by fairly conventional flagging, although the time spent at each site was not standardized. The St. John collection was obtained by a combination of active and passive surveillance; ticks were obtained by flagging backyard vegetation with a white hand towel, removing ticks from flowers harvested in the backyard, and collecting ticks from the household cat. The Rothesay and Hampton collections were obtained by passive surveillance; ticks were removed from household pets and humans following daily inspections after exposure to the same backyard or neighbourhood areas. Information on the number of site visits and number of sites is provided in Table 1 and Table 2. Environmental, landscape, and wildlife tick control measures were not in use in any of the regions surveyed. No ethics approval was required as the role of the humans in this study was to provide access to the ticks and information about ticks and humans were not the focus of the research.

### 2.4. Comparison of Tick Surveillance Strategies

Comparisons were made between the community, academic, and public health site visits that occurred between May 1 and September 30, 2014 in the greater St. John region which includes the communities of St. John, Rothesay, Quispamsis, and Hampton in southwestern New Brunswick, a Canadian Atlantic province. This region spans approximately 40 × 40 km and is within the Fundy coastal ecoregion, so it experiences similar climate, geography, and wildlife throughout its territory.

### 2.5. Tick Species Identification 

Upon arrival in the laboratory, ticks were morphologically identified as species according to Keirans and Litwak (1989) [28], then stored frozen at −20 °C for molecular analysis to assess the presence of *Borrelia* DNA. The results of this testing were returned to the tick donors.

### 2.6. DNA Extraction and Nested PCR 

DNA extraction was performed in a biological safety cabinet in a room separate from PCR and DNA analyses as described by Patterson et al. (2017) [29]. The detection of *Borrelia* in ticks was based on the amplification of two *B. burgdorferi* genes, *Flagellin B* (*FlagB*) and *Outer surface protein A* (*OspA*) by nested PCR, as described by Patterson et al. (2017) [29]. The primers used were: *OspA*outR: 5′-CAACTGCTGACCCCTCTAAT-3′, *OspA*outF: 5′-CTTGAAGTTTTCAAAGAAGAT-3′, *OspA*inR: 5′-TTGGTGCCATTTGAGTCGTA-3′, *OspA*inF: 5′-ACTTGATTAGCCTGCGCAAT-3′, *FlagB*outR: 5′-TTCAATCAGGTAACGGCACA-3′, *FlagB*outF: 5′-ACTTGATTAGCCTGCGCAAT-3′, *FlagB*inR: 5′-AGCTGAAGAGCTTGGAATGC-3′, *FlagB*inF: 5′-TCATTGCCATTGCAGATTGT-3′. The annealing temperatures were 55 °C and 58 °C for the first and second rounds, respectively, for both genes.

## 3. Results

During the spring and summer of 2014 (May–September), public health, academic, and citizen science tick surveillance projects were conducted in New Brunswick, Canada (Table 1). Initially, academic researchers already engaged in active tick surveillance were approached by community members interested in monitoring their local areas for ticks, and community members joined the academic researchers for 16 of the 66 academic tick drags conducted across the province. Additionally, some community members chose to monitor ticks independently and simply used academic researchers as resources for tick identification and testing. During the same period, a public health surveillance project was conducted in the province.

Some of the community-initiated surveillance efforts were discontinued after one or a few field collections (Table 1—health center, recreational, forestry lot collections). However, four of the community-initiated surveillance efforts continued over multiple years and encompassed many individual collections (Table 1—St. John, Nova Scotia, Hampton, Rothesay). Of these surveillance initiatives, three of the community collections (St. John, Hampton, Rothesay) overlapped spatially and temporally with a subset of the academic and public health site visits, offering the opportunity to compare surveillance strategies and tick recoveries (Table 2). 

**Figure 1:**
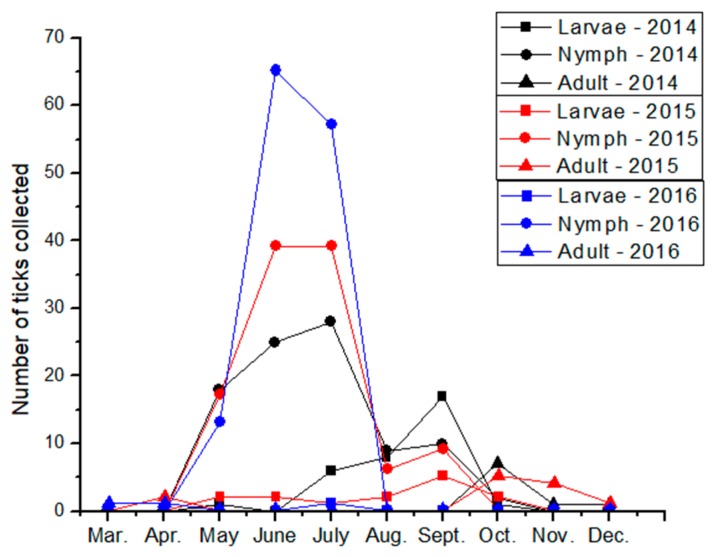
Monthly totals of *Ixodes scapularis* recoveries from the St. John collection, collected from one backyard site by passive surveillance every 2–3 days between 2014 and 2016.

**Figure 2:**
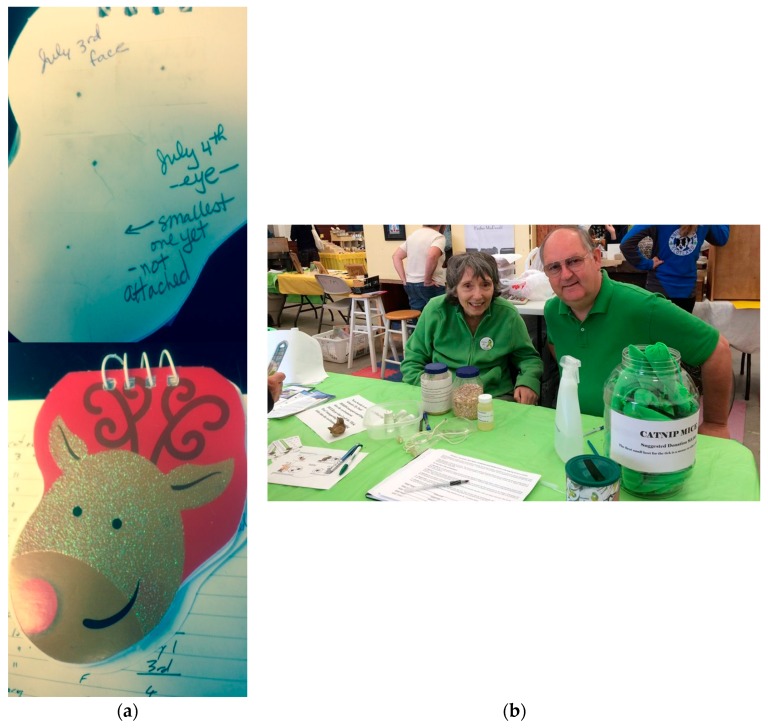
Community-initiated tick bite prevention education. (**a**) Ticks collected by a community member, taped to a notebook for display in schools (St. John collection); (**b**) Community members Brenda Sterling-Goodwin and Steve Goodwin at a Lyme awareness–tick education table at the New Glasgow, Nova Scotia, farmer’s market in 2015. The containers in the center of the table contain ticks of different species and life stages obtained from two of the community-initiated tick surveillance collections described here (Nova Scotia and Hampton collections).

The community-initiated efforts differed from the academic and public health surveillance efforts in a number of ways, including the criteria for surveillance location, the area surveyed, the number of site visits, the sampling effort, and the sampling methodology. While research teams sampled each location only once for 3 person-hours per site, in some cases (St. John and Hampton collections) the same site was sampled by the same collector daily or every few days from early spring to late fall over the course of three years. The Nova Scotia and Rothesay collections involved a broader opportunistic approach where different “likely” regions within convenient distance of the collector’s home were sampled on a daily, weekly, or biweekly schedule (Nova Scotia), or on a less frequent schedule (Rothesay). The areas selected for surveillance by the citizen scientist tick collectors were areas of concern for the collectors, their families, or communities, whereas academic or public health researchers tend to select sites to answer specific research questions. Research surveillance seeks to standardize search effort, area surveyed, and tick collector expertise. In contrast, these parameters varied for the citizen science collectors depending on the weather, terrain, prior recoveries, collector interest, collector visual acuity, collector health, and many other variables. Nevertheless, these collections all have value. These collections generate ticks that are themselves of value (Table 1), they generate data on the presence of ticks (Table 2, Figure 1), and they promote greater community awareness of ticks (Figure 2).

The St. John collection is remarkable for the very careful and frequent monitoring of a small site (family backyard) which allowed recovery of ticks at multiple life stages, including larval and nymphal ticks, as well as adults. This intensive sampling of one location also allowed the mapping of seasonal emergence of the different life stages (Figure 1), information that is generally not available from surveillance efforts using standard methodology. This type of intensive one-site sampling also lends itself to analysis of climactic factors, as described by Garcia-Marti et al. [25]. The region sampled in this collection, and the other sustained collections, were considered endemic or suspected endemic and so would not otherwise be eligible for surveillance by regional public health officials. Interestingly, tick abundance increased over the three years of intensive monitoring (Figure 1). Although this might represent improved tick surveillance methodology, the number of ticks in this collection and the recovery of larvae each year suggest that the surveillance was meticulous. This may suggest that the risk of tick-vectored disease is dynamic, even in endemic areas. The Nova Scotia collection is remarkable for the sheer number of ticks collected, although primarily adults were selected for collection. This collection also features careful notes on microclimate and vegetation conducive to tick recovery (data not shown), which is of considerable practical interest to residents of the area. 

A subset of the ticks recovered from the university surveillance efforts, the St. John, Nova Scotia, Hampton, and Rothesay collections were tested for *B. burgdorferi* infection by nested PCR. From the university collection, 0/6 (0%) tested positive for both genes (*OspA* and *FlagB*). From the St. John, Nova Scotia, Hampton, and Rothesay collections, 2/13 (15%), 6/20 (30%), 5/70 (7%), and 0/27 (0%) were positive, respectively, for both genes (*OspA* and *FlagB*). 

In addition to providing collected ticks and associated collection data to researchers, two of the community members have been very active in displaying their collections in their community and all have been strong local advocates for tick bite preventative behaviours, helping the public appreciate the presence, abundance and small size of ticks, hence the need for careful tick checks of children, adults, and pets. These activities have included showing the collected ticks at schools, farmer’s markets, and other community and social gatherings (Figure 2). By having these activities initiated within the community by trusted community members, these initiatives are a powerful means to raise public awareness of the risk of tick-borne diseases in the local area. 

## 4. Discussion

### 4.1. Advantages of Citizen Science and Community–Researcher Partnerships for Tick Surveillance

As part of a broader academic mandate to support and engage with communities, academic tick researchers partnered with community members for both joint and independent tick collection. Some of these community members joined the academic tick drags to observe the standard surveillance methodology, while others chose to monitor ticks based on a methodology of convenience or obtained from internet resources, and simply used academic researchers as a resource for identification and testing. Of these citizen science initiatives, relatively few were sustained, as reported by others, even in the context of a very well supported citizen science tick surveillance program in Holland [25]. However, four community collectors continued to monitor ticks over a multi-year period, one performing a remarkable 146 submission days (St. John collection) and another recovering over 1400 ticks (Nova Scotia collection). During the spring and summer of 2014, public health officials and designates also conducted active surveillance in New Brunswick [27]. As some of these collections overlapped geographically and temporally, this allowed the comparison of the relative outcomes of each type of collection approach (Table 2).

The public health and academic surveillance projects both used very similar methodologies: active surveillance by a standardized 3 person-hours tick dragging at each site, but with only one site visit. This approached recovered 0–0.2 ticks/site (Table 2). Within the same region, the citizen science tick collectors found 0.15–2.5 ticks per site visit (Table 2). Despite the range in tick recoveries, which could be due to geography or collector experience and skill, the community surveillance efforts clearly outperformed both the academic and public health tick surveillance initiatives in numbers of ticks recovered, both in total ticks recovered and per site visit. 

The enhanced tick recovery by the community members could be due to any of the differences in methodology between community tick collectors and academic or public health researchers. One likely contribution to enhanced recovery is repeated site visits. Recovery of ticks by active surveillance is well known to be inefficient and dependent on a host of variables, both abiotic and biotic [19,20]. Multiple field samplings can mitigate the effect of these variables. Repeated site visits is an approach favoured by citizen scientists for its convenience and responsiveness to local concerns, but it is an approach that is logistically challenging for research and public health surveillance. In addition to repeated site visits, the community tick collections described here used primarily passive rather than active tick surveillance. However, even if passive surveillance is the reason for the enhanced recovery by the citizen scientists, both approaches had zero recovery days suggesting that it is the repeated visits that make these citizen collections so effective. The repeated site visits are also what makes these collections so valuable.

Access to the high-density, local scale, longitudinal tick surveillance data provided by community-based active tick surveillance is not otherwise readily available. This type of data allows investigation of interplay between local microclimate, local reservoir species abundance, and the variety of other biotic and abiotic factors that affect tick populations but are not well understood. This work, Seifert et al. (2016) [24],, and Garcia-Marti et al. (2017) [25] all document the microheterogeneity of tick recoveries: simultaneous tick drags conducted only a few meters apart or conducted only a few days apart can yield very different recoveries. Yet, as demonstrated by Garcia-Marti et al. (2017) [25], the high-density longitudinal data generated by volunteer community members can generate valuable data that can start to address these questions and even provide sufficient data for the construction of a model that can predict daily tick activity at a national level. 

The ticks recovered are also a research resource in themselves; the ticks recovered by the community surveillance initiatives described here have been used for a variety of research projects ranging from novel tick diagnostics to tick microbiome analysis [30]. Further, with the exception of one larva recovered in the public health surveillance initiative [27], the St. John collection yielded the most plentiful supply of immature ticks. As the immature life stages are often of prime interest to researchers, this demonstrates the value of the meticulous and intensive tick surveillance conducted by community members.

Finally, the most important advantage of tick surveillance is the increased community awareness and commitment to tick bite prevention practices, which would be expected to result in decreased risk of tick borne disease in that community (Figure 2). The educational value of researcher – community partnership is demonstrated by Seifert et al. (2016) [24] who quantified the increased awareness of tick bite prevention strategies, consisting in tick checks, the use of protective clothing and repellents, and the awareness of signs of early infection, in high school students engaged in citizen science tick surveillance. Resistance or indifference to conventional public health messaging is an ongoing problem that can be effectively and inexpensively circumvented by partnering with trusted community leaders [31]. A public health poster on a doctor’s office wall can easily be ignored; your neighbour showing you a tick retrieved from the head of their child will have much greater emotional impact. This seems not only intuitively reasonable, but also strongly suggested by anecdotal evidence as shown in Figure 2. Finally, the low rate of tick recoveries during many tick drags can also be useful in countering “tickophobia” and provide an increased sense of personal security outdoors and empowerment that can lead to increased use of outdoor areas for recreation [24]. 

### 4.2. Disadvantages

Community members provided much more extensive and detailed tick collections, with attendant high-density information, than either recourse-limited academic or public health tick surveillance initiatives (Table 1 and Table 2). However, despite the value of this dataset and the other important advantages of community engagement in tick surveillance through citizen science, there are disadvantages to this approach. In this study, all of the sustained collections were by individuals who used their own methodology for tick collection rather than a standard methodology. Whether this is coincidental or due to trained researchers having a higher tolerance for the tedium intrinsic to the standard methodology is unclear. Seifert et al. (2016) [24] also noted that the generation of innovative approaches increased tick recovery, and that this innovation was coupled to engagement in the surveillance initiative. Unsuccessful citizen science initiatives are characterized by a top-down attempt to get community members to perform activities to research standards. After an initial phase of enthusiasm, community engagement evaporates under pressure from the daily demands of life [31]. In contrast, successful citizen science projects are collaborative, often iterative, in adapting a methodology to volunteers’ need, interest, and time. In this study, their average per site tick recovery and the recoveries of immature stages by the different community tick collectors varied more than tenfold. The different recoveries of adults and immature stages presumably reflect a combination of geographic considerations, sampling effort, and collector ability. The latter may have a very strong influence on the recoveries of the immature stages, considering that all sites surveyed were endemic, thus including all life stages, and were surveyed throughout the year. However, it is important to note that this is not a weakness restricted to citizen scientist collectors; academic and public health researchers also had limited recovery of the immature stages. Regardless of the cause of this variation, variation in data collection methodologies and quality is a normal aspect of many studies that can be managed through the introduction of appropriate internal quality control monitoring and post-collection data analysis. Using the number of site visits and of immatures stages recovered as indicators of collection integrity and quality would be convenient and obvious internal quality assessment measures. Garcia-Marti (2017) et al. [25] successfully used citizen science-generated data to generate a nation-wide predictive model for tick abundance by using such internal quality control steps, although at the cost of discarding data from many of their collections. Similarly, Kampen et al. (2015) [32] and Bates et al. (2015) [33] noted similar considerations in their citizen science invertebrate surveillance studies. The participation of community members greatly increases the scope of the surveillance efforts as long as project design and analysis are adaptive and a strong communication with community members is maintained. Indeed, the use of different methodologies by different investigators working on the same problem is the norm in science and does not prevent the comparison of results or scientific progress, so this aspect of citizen science, while requiring some effort on the part of the research partners, does not negate the value of the data generated by citizen science projects or the associated value of these initiatives.

**Figure 3:**
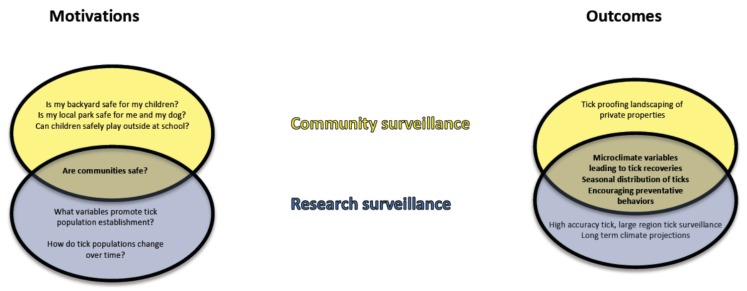
Overlap in motivations and outcomes of community- and research-initiated tick surveillance efforts. Community- and research-initiated efforts differ in the scale and expense of their investigation but, as both are designed to enhance community safety, research and community outcomes are highly overlapping.

A related consideration, although specific only to this study, is that the two most productive collections were from areas already identified as endemic and the other two from suspected endemic areas. The focus of researcher-initiated surveillance efforts is on regions of expanding and newly establishing tick populations so, in this sense, surveillance is only useful when directed at areas where tick populations are not endemic. In contrast, local concerns tend to be high in areas of high tick density—in these areas, people are more likely to encounter ticks, and concerns as to whether playing in a backyard or school playground is safe for children are a very immediate and powerful motivator (Figure 3). Additionally, collecting in endemic areas provides a greater “reward” in that the probability of finding ticks is greater. However, the risk to human health is greatest in endemic areas so, while research and community motivations are disparate, they do overlap and the outcomes of both community-based and public health- and academic-based surveillance efforts overlap considerably and yield valuable information (Figure 3). 

### 4.3. Benefits and Applications of Citizen Science Tick Surveillance Projects

In addition to the overlapping motivations and outcomes of tick surveillance, tick surviellance lends itself to community–researcher partnerships; repeated sampling of small sites is too expensive for academic or public health researchers, but the molecular analysis required to assess the infection status of the ticks requires sophisticated molecular genetic expertise not otherwise available to community members. Citizen science projects such as the community–academic tick surveillance partnerships described here lay the foundation for transmission of scientific knowledge to the public and allow communities can act on this information. A collaborative partnership between academic partners and schools was effective in encouraging students to practice tick bite prevention strategies, as described by Seifert et al. (2016) [24]. Various patient advocacy groups, for example the Global Lyme Alliance, working in partnership with educators has designed teaching modules focusing on tick awareness education for students of all ages (globallymealliance.org) which could be readily introduced into educational programs. Further, the high-density data generated by these partnerships can be used to model seasonal, even daily, tick activity estimates, as described by Garcia-Marti (2017) et al. [25]. This information could be used to inform those using wilderness areas recreationally or working in forested areas of the local and seasonal risk of tick encounters, in much the same way that forest fire risk is advertised, or flu season activity is publicly posted. Park maintenance activities, such as mowing and watering, could also be seasonally modified to reduce public risk. The Dutch citizen science website Tekenradar [Tick radar] (www.tekenradar.nl) posts tick risk maps, as does the Tick Encounter Resource Center, an initiative of the University of Rhode Island that actively engages the public in tick awareness and monitoring (www.tickencounter.org). Thus, the success of the community-driven tick surveillance efforts documented here emphasizes the value of partnering with community members in citizen science tick surveillance.

## 5. Conclusions

By adapting our tick surveillance methodology to incorporate contributions and participation from community volunteers in response to local and individual interests and needs, we have maintained useful submissions over a multi-year period. Community-initiated tick surveillance provides information complementary to that from standardized tick surveillance, and can be thus used to address research questions not otherwise accessible from broad-scale surveillance. Most importantly, citizen science initiatives are ideally suited to promote local knowledge, foster trust, and translate this knowledge into effective preventative behaviours needed to protect the public in the face of the increased risk of tick-vectored diseases.

## Figures and Tables

**Table 1:** Tick recoveries from academic and community-initiated surveillance efforts.

Collection	Collection Type	Year	Location	Number of Sites	Number of Site Visits	Collection Method	*Ixodes scapularis*					Other Ticks
							Larvae	Nymph	Adult Female	Adult Male	Total	
Mt. Allison	academic	2014	NB	66	1	active	0	0	6	3	9	
Sackville Health Center	community/academic	2014	Sackville, NB	1	1	active	0	0	0	0	0	*I. cookei* (3 adults)
Recreational	community/academic	2014	Kejimkujik Park, NS	1	3	active	0	0	0	0	0	*Dermacentor variabilis* (6 adults)
NB Forestry	community/academic	2015	Fredericton, NB	7	1	active	0	0	0	0	0	*D. variabilis* (4 adults)
St. John	citizen	2014	Millidgeville, NB	1	38	passive	33	82	2	0	117	
		2015		1	53		13	108	11	1	133	
		2016		1	26		1	137	2	0	140	
Nova Scotia	citizen	2015	Lunenburg, NS	2	6	active	0	0	93	73	166	*D. variabilis* not enumerated
		2016		2	12		0	1	304	220	525	
		2017		2	16		0	0	388	328	716	
Hampton	citizen	2012	Hampton, NB	1	200 ^a^	passive	0	0	13	0	13	
		2013		1	200 ^a^		0	0	12	0	12	
		2014		1	200 ^a^		0	0	15	0	15	
		2015		1	200 ^a^		0	0	3	0	3	
		2016		1	200 ^a^		0	0	5	0	5	
Rothesay	citizen	2014	Rothesay, NB	1	6	passive	0	0	3	3	6	
		2016		1	8		0	0	5	9	14	

^a^ Estimated number of site visits.

**Table 2:** Comparison of effectiveness of tick recovery per site visit by different groups performing tick surveillance in the same area during the same time period.

Collection	Total of Ticks Recovered ^a^	Number of Sites ^b^	Number of Visits/Site	Average Tick/Site Visit
Public Health	0	8	1	0
Academic	7	38	1	0.2
Citizen (St. John)	94	1	38	2.5
Citizen (Hampton)	15	1	100 ^c^	0.15
Citizen (Rothesay)	6	1	8	0.75

^a^ Ticks recovered from May 1 to September 30, 2014 from collections within the St. John regional area in southwestern New Brunswick, Canada; ^b^ a site is defined as a location separated by >200 m from another location; ^c^ Estimated number of site visits.
